# Prophylactic use of levosimendan in pediatric patients undergoing cardiac surgery: a prospective randomized controlled trial

**DOI:** 10.1186/s13054-019-2704-2

**Published:** 2019-12-30

**Authors:** Anbiao Wang, Chaomei Cui, Yiou Fan, Jie Zi, Jie Zhang, Guanglai Wang, Fang Wang, Jun Wang, Qi Tan

**Affiliations:** 1 Intensive Care Unit (ICU), Department of Cardiac Surgery, Provincial Hospital Affiliated to Shandong University, No. 9677 Jingshi Road, Jinan, 250021 China; 2 Intensive Care Unit (ICU), Department of Cardiac Surgery, Provincial Hospital Affiliated to Shandong First Medical University, Jinan, 250021 China; 3 Department of Toxicological and Functional Test, Centers for Disease Control and Prevention of Shandong, Jinan, 250014 China; 4 Department of Pharmacy, Shandong Provincial Hospital Affiliated to Shandong University, Jinan, 250021 China

**Keywords:** Levosimendan, Pediatric, Cardiac surgery, Low cardiac output syndrome, Safety

## Abstract

**Background:**

The administration of levosimendan prophylactically to patients undergoing cardiac surgery remains a controversial practice, and few studies have specifically assessed the value of this approach in pediatric patients. This study therefore sought to explore the safety and efficacy of prophylactic levosimendan administration to pediatric patients as a means of preventing low cardiac output syndrome (LCOS) based upon hemodynamic, biomarker, and pharmacokinetic readouts.

**Methods:**

This was a single-center, double-blind, randomized, placebo-controlled trial. Patients ≤ 48 months old were enrolled between July 2018 and April 2019 and were randomly assigned to groups that received either placebo or levosimendan infusions for 48 h post-surgery, along with all other standard methods of care. LCOS incidence was the primary outcome of this study.

**Results:**

A total of 187 patients were enrolled, of whom 94 and 93 received levosimendan and placebo, respectively. LCOS incidence did not differ significantly between the levosimendan and placebo groups (10 [10.6%] versus 18 [19.4%] patients, respectively; 95% confidence interval [CI] 0.19–1.13; *p* = 0.090) nor did 90-day mortality (3 [3.2%] versus 4 [4.3%] patients, CI 0.14–3.69, *p* = 0.693), duration of mechanical ventilation (median, 47.5 h and 39.5 h, respectively; *p* = 0.532), ICU stay (median, 114.5 h and 118 h, respectively; *p* = 0.442), and hospital stay (median, 20 days and 20 days, respectively; *p* = 0.806). The incidence of hypotension and cardiac arrhythmia did not differ significantly between the groups. Levels of levosimendan fell rapidly without any plateau in plasma concentrations during infusion. A multiple logistic regression indicated that randomization to the levosimendan group was a predictor of LCOS.

**Conclusions:**

Prophylactic levosimendan administration was safe in pediatric patients and had some benefit to postoperative hemodynamic parameters, but failed to provide significant benefit with respect to LCOS or 90-day mortality relative to placebo.

**Trial registration:**

Name of the registry: Safety evaluation and therapeutic effect of levosimendan on the low cardiac output syndrome in patients after cardiopulmonary bypass. Trial registration number: ChiCTR1800016594. Date of registration: 11 June 2018. URL of trial registry record: http://www.chictr.org.cn/index.aspx

## Background

The 2019 report of cardiovascular disease in China indicated that there are roughly 2 million individuals suffering from congenital heart disease (CHD), with over 110,000 pediatric patients undergoing surgical treatment for this condition annually. Given that pediatric patients have a very physiologically limited contractile reserve, they are at a high risk of marked reductions in ventricular performance during cardiac surgery as a consequence of factors such as hypoxia, acidosis, ischemia-reperfusion injury, neurohormone-mediated activation, or systemic inflammation [1, 2]. These risk factors can lead to 3~14% of patients that undergo surgery with cardiopulmonary bypass (CPB) suffering from heart failure or low cardiac output syndrome (LCOS), while these rates can be as high as 25% in pediatric patients and are linked to a 15-fold rise in morbidity and mortality following surgical treatment of CHD [3–5].

As the consequences of LCOS can be fatal, determining the optimal treatment strategy for these pediatric patients remains a daunting task. The administration of specific pharmaceutical agents is essential to achieving satisfactory pre-, peri-, and postoperative outcomes for LCOS. However, specific guidelines regarding the safe and effective use of drugs for the prevention and treatment of LCOS in infants and children are lacking. Catecholamine and milrinone remain the current traditional approach to treating LCOS [6]. Catecholamines can provide a valuable short-term benefit, but these benefits are constrained by adrenergic receptor downregulation and excessive chronotropy as doses are escalated. Milrinone has been prophylactically administered to patients, helping to prevent postoperative LCOS and associated clinical symptoms, but this drug also causes well-characterized and potentially serious side effects due to increases in myocyte cyclic adenosine monophosphate levels, leading to tachycardia, elevated myocardial oxygen consumption, and even myocardial necrosis [7].

The inotropic and lusitropic effects of levosimendan at low concentrations (nM) have been attributed to Ca^2+^ sensitization and phosphodiesterase3 inhibition mechanisms [8]. As it acts in a very specific manner, levosimendan has been found to achieve satisfactory preconditioning and positive inotropic effects without Ca^2+^ overload, thereby improving survival rates among adult heart failure patients [9, 10]. Furthermore, levosimendan has been linked to higher CPB weaning rates, lower periprocedural myocardial infarction rates, and decreased lactate levels owing to superior tissue perfusion as compared with placebo [11, 12], dobutamine [13], or milrinone [14]. Although many small-scale trials have suggested that levosimendan can provide substantial benefit to patients, large-scale comparative trials have not been able to reproduce this effect [15]. Evidence-based medicine in pediatric cardiology is a particular challenge, and levosimendan is still used as an off-label drug for pediatric patients in most counties, potentially due to insufficient supportive research data. At present, levosimendan pharmacokinetics in pediatric patients is not well-documented. However, given that pediatric myocyte contractility is more Ca^2+^-dependent than in adults, and that small-scale studies have shown great promise with respect to the mechanism of action of levosimendan, further assessment of whether this drug can prevent or treat LCOS in pediatric patients is warranted.

In the present study, we aimed to assess the safety and efficacy of prophylactically administering levosimendan to pediatric patients undergoing cardiac surgery with CPB, working under the hypothesis that prophylactic administration of this compound for 48 h after surgery may help to reduce LCOS incidence. Importantly, we have for the first time implemented valuable invasive hemodynamic monitoring and described the pharmacokinetics of levosimendan in the context of cardiac surgery in pediatric patients. This was a single-center pilot study designed with the goal of supporting a consecutive multicenter trial.

## Materials and methods

### Trial design and oversight

This was a single-center, prospective, randomized, placebo-controlled trial that received approval from the Ethics Committee of the Provincial Hospital affiliated to Shandong University (approval number 2018-019). In addition, this study was registered with the National Clinical Trial Center (ChiCTR1800016594). Patients treated in this trial purchased the drug at full cost, and trial design, data collection, subsequent analyses, and manuscript submission were not influenced by the drug manufacturer or the funding agencies supporting this trial. All authors affirmed that the data and analyses in this trial were accurate and complete and that the trial was conducted in a manner consistent with the study protocol. The Shandong Centers for Disease Control and Prevention served as a neutral third party which supported data management and quality control. The School of Public Health of Shandong University performed all statistical analyses for this study.

### Study participants

The parents of all pediatric patients scheduled to undergo cardiac surgery provided written informed consent. Eligible patients had to be ≤ 48 months old and scheduled to undergo cardiac surgery with the use of CPB. In addition, these patients were in categories 2~5 according to the Risk Adjustment for Congenital Heart Surgery (RACHS) method (as reported in Additional file 1). Full inclusion/exclusion criteria are shown in Additional file 1: Table S1.

### Randomization and blinding

Eligible patients were assigned at random to receive either levosimendan or placebo infusions (at a 1:1 ratio) following the postoperative entry into the ICU. A computer-generated, permuted block sequence stratified according to the trial center was used to support the study randomization. Sequentially numbered opaque envelopes containing participant assignment groups were sealed, with all physicians, patients, outcome assessors, and research staff being blinded to the patient treatment assignments.

### Study interventions

All patients were at random assigned to receive a preparation of levosimendan (QiLu Medicine Corporation, China) or a placebo control, with these preparations being prepared by dedicated trial personnel such that patients and physicians were unaware of which treatment a given patient was received. For levosimendan preparation, 12.5 mg of the drug (5 mL) was dissolved into 45 mL of 5% glucose. As a placebo, a yellow-colored solution of vitamins without any relevant cardiovascular effects but visually identical to levosimendan was instead used, shown in Additional file 1: Figure S1. Patients received a continuous 0.05 μg/kg/min infusion of their indicated treatment as quickly as possible following surgery. Infusions were continued for 48 h, adjusting infusion rate according to adverse events. Attending physicians were given full discretion to administer any additional medications to the patients as appropriate, including other inotropes and vasopressors. Concomitant nesiritide use was not permitted.

### Data collection and follow-up

We collected baseline, intraoperative, postoperative, clinical, and safety outcome data of each patient. Potential postoperative conditions of patients were monitored including acute renal injury necessitating dialysis or pneumonia. Myocardial enzymology analyses included troponin T (TNT), creatine phosphokinase-MB (CK-MB), and NT-proBNP; invasive hemodynamic parameters were determined via the pressure recording analytical method (PRAM) including heart rate (HR), systolic atrial pressure (SAP), cardiac index (CI), systemic vascular resistance index (SVRI), maximum pressure gradient (dp/dt_MAX_), and cardiac cycle efficiency (CCE) [16]. CCE is a novel indicator that describes hemodynamic performance in terms of energy expenditure (as reported in Additional file 1). Vasoactive-Inotropic Score (VIS) is calculated as follows: dopamine (μg/kg/min) + dobutamine (μg/kg/min) + [100 × epinephrine (μg/kg/min)] + [10 × milrinone (μg/kg/min)] + [10,000 × vasopressin (U/kg/min)] + [100 × norepinephrine (μg/kg/min)] [17]. Patients underwent 90 days of post-surgical follow-up, with survival and rehospitalization status during this period being recorded.

### Pharmacokinetic assessments

At 6, 12, 24, 48, 72, and 120 h after initiating levosimendan dosing, venous blood samples were collected in chilled tubes that contained K_3_EDTA to measure plasma levosimendan concentrations, while collections at 6, 12, 24, 48, 72, 120, and 168 h after initiating levosimendan dosing were used for measuring the concentrations of OR-1855 and OR-1896 metabolites in the plasma.

High-performance liquid chromatography (HPLC) with ultraviolet detection was used to detect levosimendan concentrations, while HPLC with tandem mass spectrometry was used to detect concentrations of the indicated metabolites of OR-1855 and OR-1896. Terminal half-life (*T*_1/2_), time to peak concentration (*T*_max_), peak concentration in the plasma (*C*_max_), area under the curve to the last measurable concentration (AUC_0−*t*_), and area under the concentration-time curve (AUC_0−∞_) were obtained.

### Study endpoints

LCOS incidence was the primary outcome of this trial. LCOS was defined as two consecutive measurements of low cardiac output (defined as a cardiac output of ≤ 2.2 L/min/m^2^, without associated relative hypovolaemia), one measurement of low cardiac output plus the use of two or more inotropes at or beyond 24 h after surgery, or the use of two or more inotropes at or beyond 24 h after surgery with the indicated reason being low cardiac output. Secondary outcomes for this trial included (a) mortality and rehospitalization within 90 days post-surgery; (b) duration of mechanical ventilation; (c) durations of ICU and hospital stay; (d) incidences of postoperative complications; (e) myocardial enzymology at 24, 48, 72, and 96 h post-surgery; (f) invasive hemodynamic parameters at 24, 48, and 72 h post-surgery; (g) VIS at 2, 24, 48, and 72 h post-surgery; and (h) post-surgical safety outcomes including hypotension, arrhythmia, and hepatorenal function.

### Statistical analysis

Sample size calculations were based upon a two-sided α error of 0.05 with 80% power. A previous randomized controlled trial was used to estimate the expected primary outcome effect size [18]. In this previous report, the control and levosimendan groups had LCOS incidence rates of 61% and 37%, respectively. As such, we made a hypothesis based upon the expected LCOS rates of 45% and 25% in the placebo and levosimendan groups, respectively, with the potential for up to 5% of participants to be lost to follow-up. Based on these calculations, we determined that 90 patients per group were needed, leading us to ultimately enroll 94 and 93 patients in the levosimendan and placebo groups, respectively.

Normally and non-normally distributed data were presented as means with standard deviations and medians with interquartile ranges, respectively, and categorical variables were presented as *n* (%). Two-tailed chi-squared tests with Yates correction were used to compare the prespecified postoperative events of interest. Student’s *t* test or Mann-Whitney *U* tests were used to compare continuous variables. Odds ratios and confidence intervals were estimated from a logistic regression model with age, BSA, sex, and RACHS as covariates. The duration of mechanical ventilation, duration of ICU stay, and duration of hospital stay were analyzed by linear regression with the same covariates. Univariate analysis and multivariate logistic regression modeling were used to assess the associations between baseline factors and LCOS incidence. Mortality at 90 days was summarized with the use of Kaplan-Meier estimates and log-rank tests. A two-sided *p* < 0.05 was the significance threshold. All the statistical analyses were performed at the School of Public Health of Shandong University (Shandong Province, China) with the use of SPSS software, version 25.0.

## Results

### Enrollment and baseline characteristics

**Fig. 1:**
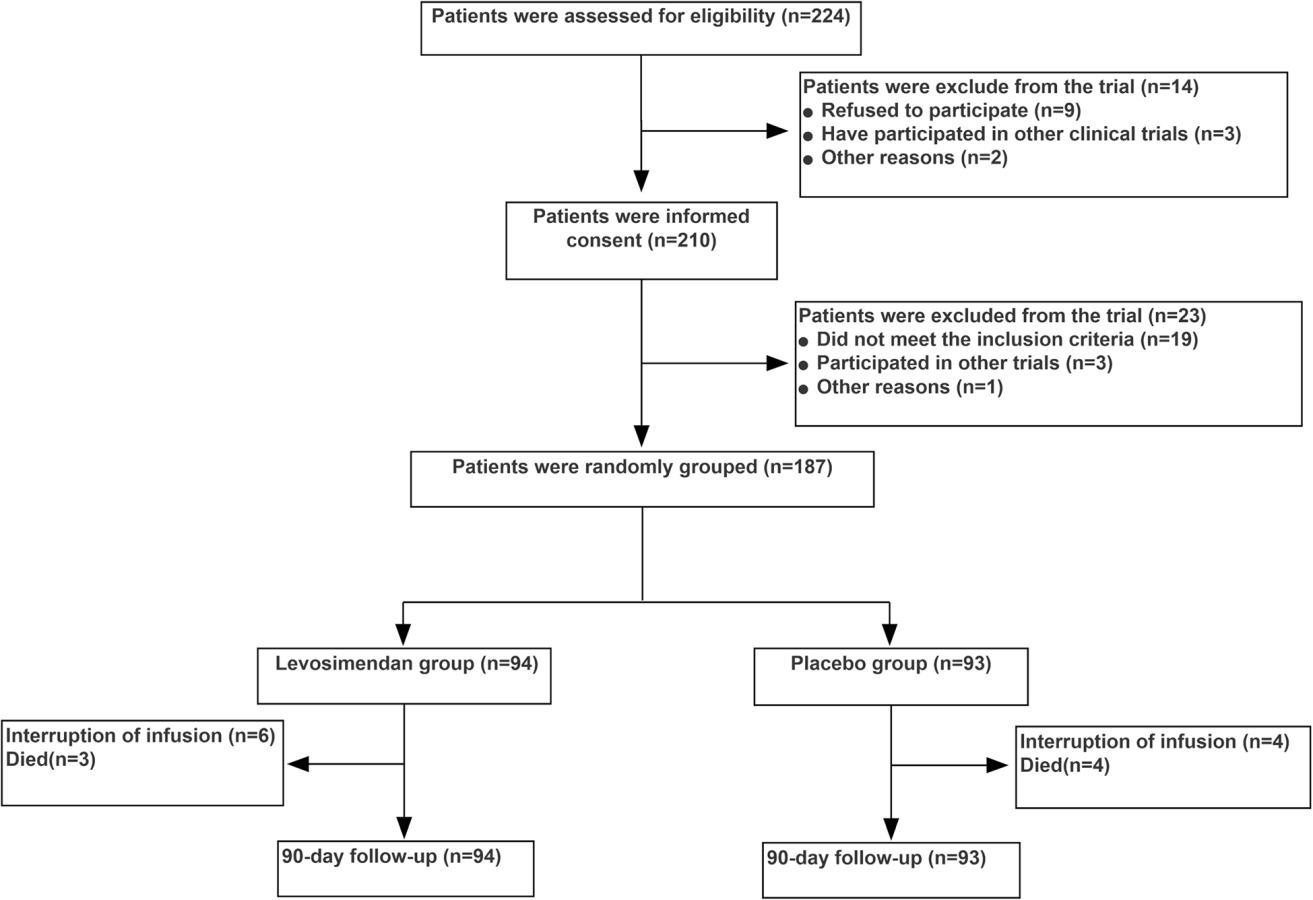
Recruitment, randomization, and analysis populations

We assessed the eligibility of 224 consecutive pediatric patients between July 2018 and April 2019, with the parents of 210 of these patients providing written informed consent. Patient randomization and follow-up are detailed in Fig. 1. All patients that survived completed the 90 days of study follow-up.

Patients in both groups had similar baseline and intraoperative characteristics, with no differences in age, gender, BMI, BSA, or RACHS between the groups, shown in Table 1. The CPB duration in the levosimendan and placebo groups was 81 ± 34 and 84 ± 30 min (*p* = 0.479), respectively, whereas the cross-clamp duration was 46 ± 23 and 51 ± 22 min (*p* = 0.163), respectively. Baseline hemodynamics and biomarkers also did not differ between the groups.

**Table 1:** Study population baseline demographic and surgical characteristics

Characteristics	Levosimendan group (*n* = 94)	Placebo group (*n* = 93)	*p* value
Age (months)^a^	5 (2,11)	7 (2,16)	0.318
Gender, female, *n* (%)	42 (44.7%)	41 (44.1%)	1.000
BMI (kg/m^2^)	14.83 ± 2.21	14.76 ± 2.13	0.870
BSA (m^2^)	0.36 ± 0.17	0.39 ± 0.19	0.248
RACHS classification, *n* (%)*			0.146
RACHS 2	53 (63.1%)	67 (72.0%)	
RACHS 3	21 (25.0%)	11 (11.8%)	
RACHS 4	9 (10.7%)	13 (14.0%)	
RACHS 5	1 (1.2%)	2 (2.2%)	
Down’s syndrome, *n* (%)	3 (3.2%)	2 (2.2%)	0.660
CPB (min)	81 ± 34	84 ± 30	0.479
Cross-clamp (min)	46 ± 23	51 ± 22	0.163

Data are means ± standard deviation (SD) for continuous variables and number of subjects (*n*) and percentage (%) for categorical variables

*BSA* body surface area, *RACHS* Risk Adjustment for Congenital Heart Surgery, *CPB* cardiopulmonary bypass, *BMI* body mass index

^a^Data are medians [Q1, Q3]

*RACHS classification was used to divide surgical procedures for congenital heart diseases into six categories of increasing predicted operative risk. The greater the score, the higher the risk associated with the procedure

### Levosimendan or placebo infusion

The average time until infusion initiation in the levosimendan and placebo groups was 2.27 ± 0.81 h and 2.39 ± 0.81 h post-surgery, respectively (*p* = 0.453; Table 2). A total of 83 and 86 patients in the levosimendan and placebo groups (88.3% and 92.5%, respectively) completed 48 h infusion course, while 3 and 2 patients in the levosimendan and placebo groups (3.2% and 2.2%, respectively) were infused < 24 h, and 8 and 5 patients in the levosimendan and placebo groups (8.5% and 5.4%, respectively) were infused for 24 to 48 h. The reasons for non-completion of levosimendan or placebo regimens are shown in more detail (as reported in Additional file 1).

**Table 2:** Administration of levosimendan or placebo

Variable	Levosimendan group (*n* = 94)	Placebo group (*n* = 93)	*p* value
Time of infusion started after surgery (h)	2.27 ± 0.81	2.39 ± 0.81	0.453
Interruption of infusion due to adverse events, *n* (%)	6 (6.4%)	5 (5.4%)	0.770
Duration of infusion, *n* (%)			
< 24	3 (3.2%)	2 (2.2%)	0.659
24–48	8 (8.5%)	5 (5.4%)	0.399
48	83 (88.3%)	86 (92.5%)	0.333

Data are means ± standard deviation (SD) for continuous variables and number of subjects (*n*) and percentage (%)for categorical variables

### Primary and secondary outcomes

**Fig. 2:**
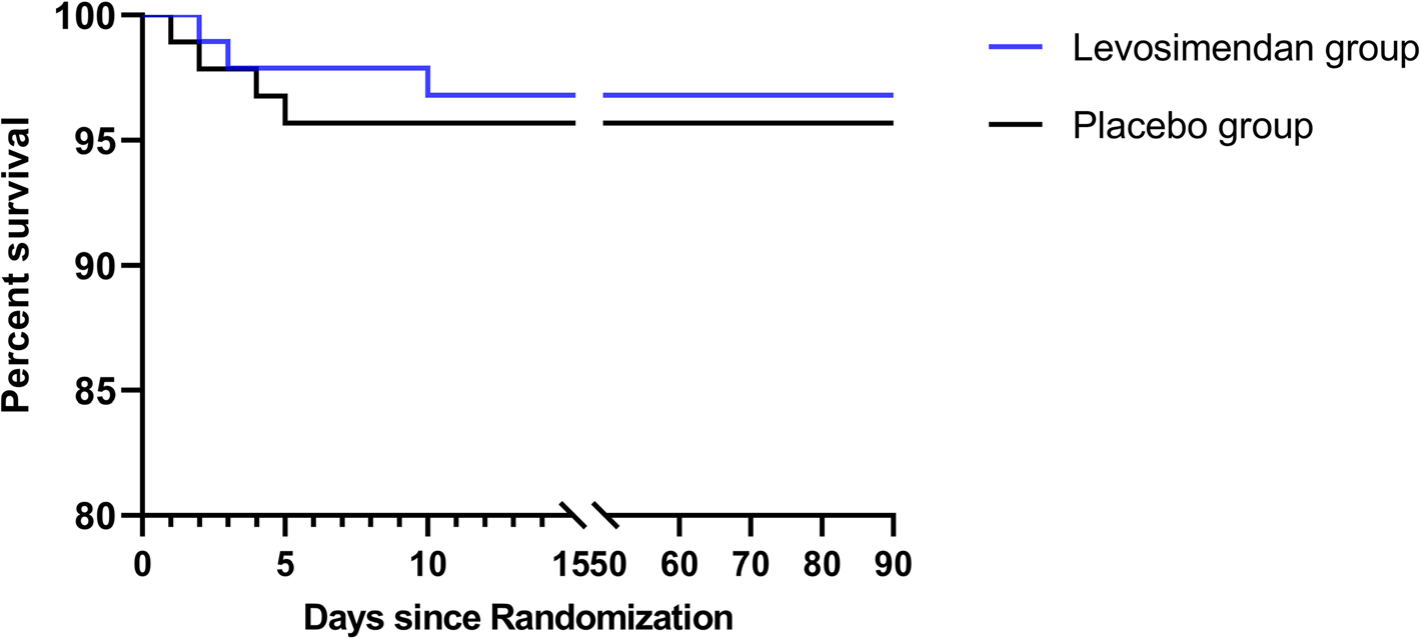
Kaplan-Meier survival estimates of mortality

LCOS incidence, which was the primary study outcome, was 10.6% (10/94) in the levosimendan group and 19.4% (18/93) in the placebo group (OR 0.46; 95% CI, 0.19–1.13; *p* = 0.09). With respect to the secondary outcomes, 900-day mortality in the levosimendan group was 3.2% (3/94) versus 4.3% (4/93) in the placebo group (OR, 0.72; 95% CI, 0.14–3.69; *p* = 0.693). Survival curves also indicated that there were no differences in between-group mortality rates over time (*p* = 0.685; Fig. 2). Rates of rehospitalization within 90 days were 3.2% (3/94) and 1.1% (1/93) in the levosimendan and placebo groups, respectively (OR 2.57; 95% CI, 0.24 to 27.33; *p* = 0.433), with no significant differences in the duration of mechanical ventilation (median, 47.5 h and 39.5 h, respectively; *p* = 0.532), ICU stay (median, 114.5 h and 118 h, respectively; *p* = 0.442), or hospital stay (median, 20 days and 20 days, respectively; *p* = 0.806), as shown in Table 3. Hemodynamic results were compiled in Table 4, and myocardial enzymology was shown in Additional file 1: Table S3. There was a significant difference between the groups with respect to the CCE hemodynamic variable following infusion, while arterial blood gas and VIS did not differ between the groups, as shown in Additional file 1: Table S4 and S5.

**Table 3:** Pre-specified clinical outcomes

Outcome	Levosimendan group (*n* = 94)	Placebo group (*n* = 93)	OR (95% CI)	*p* value
Primary outcome				
Incidence of LCOS, *n* (%)	10 (10.6%)	18 (19.4%)	0.46 (0.19–1.13)	0.090
Second outcomes				
90-day mortality, *n* (%)	3 (3.2%)	4 (4.3%)	0.72 (0.14–3.69)	0.693
Rehospitalization at 90 days, *n* (%)	3 (3.2%)	1 (1.1%)	2.57 (0.24–27.33)	0.433
Duration of mechanical ventilation (h)	47.5 (21.4, 96.0)	39.5 (18.0, 97.3)	–	0.532
Duration of ICU stay (h)	114.5 (72.38, 189)	118 (69, 200.25)	–	0.442
Duration of hospital stay (days)	20 (17, 27)	20 (17, 26)	–	0.806
Sepsis, *n* (%)	4 (4.3%)	6 (6.5%)	0.64 (0.18–2.36)	0.512
Pneumonia, *n* (%)	10 (10.6%)	12 (12.9%)	0.80 (0.33–1.96)	0.631
AKI, *n* (%)	1 (1.1%)	2 (2.2%)	0.49 (0.04–5.49)	0.557
Renal replacement therapy, *n* (%)	1 (1.1%)	1 (1.1%)	0.99 (0.06–16.05)	0.991
Wound infection, *n* (%)	1 (1.1%)	1 (1.1%)	0.99 (0.06–16.05)	0.997
Safety outcomes				
Hypotension during infusion, *n* (%)	2 (2.1%)	2 (2.2%)	1.69 (0.39–7.27)	0.476
Arrhythmias during infusion, *n* (%)	4 (4.26%)	3 (3.23%)	1.20 (0.35–4.08)	0.773

Data are medians [Q1, Q3] for continuous variables and number of subjects (*n*) and percentage (%) for categorical variables. Differences between the percent values are given in percentage points, thus potentially not summing to the expected values as a consequence of rounding. Other variable differences are in the indicated units

*CI* confidence interval, *OR* odds ratio, *LCOS* low cardiac output syndrome, *AKI* acute kidney injury

**Table 4:** Hemodynamic analysis

Outcome	Levosimendan group (*n* = 94)	Placebo group (*n* = 93)	*p* value
Before surgery			
HR (bpm)	127.9 ± 12.5	130.2 ± 11.2	0.185
SBP (mmHg)	81.1 ± 5.8	80.3 ± 7.3	0.403
CI (L/min/m^2^)	3.42 ± 0.58	3.42 ± 0.51	0.857
SVRI (dyne s/m^2^ cm^5^)	1134.4 ± 170.5	1079.5 ± 158.7	0.384
dp/dt (mmHg/ms)	0.86 ± 0.21	0.89 ± 0.23	0.265
CCE (units)	0.38 (0.30, 0.42)	0.35 (0.24, 0.41)	0.235
2 h after surgery			
HR (bpm)	139.7 ± 13.0	138.3 ± 13.7	0.464
SBP (mmHg)	79.4 ± 7.7	79.3 ± 7.9	0.898
CI (L/min/m^2^)	2.60 ± 0.69	2.64 ± 0.78	0.701
SVRI (dyne s/m^2^ cm^5^)	1899.2 ± 711.5	1836.5 ± 856	0.626
dp/dt (mmHg/ms)	0.66 ± 0.29	0.65 ± 0.24	0.774
CCE (units)	− 0.52 (− 0.89, − 0.16)	− 0.69 (− 1.02, − 0.30)	0.066
24 h after surgery			
HR (bpm)	139.2 ± 14.7	135.3 ± 14.3	0.073
SBP (mmHg)	77.1 ± 6.4	76.0 ± 9.9	0.384
CI(L/min/m^2^)	2.63 ± 0.99	2.46 ± 0.59	0.199
SVRI (dyne s/m^2^ cm^5^)	1746.0 (1481.0, 2073.0)	1765.0 (1430.0, 2068.0)	0.466
dp/dt (mmHg/ms)	0.62 (0.47,0.81)	0.49 (0.38, 0.70)	0.013
CCE (units)	− 0.37 (− 0.75, − 0.09)	− 0.54 (− 1.0, − 0.19)	0.043
48 h after surgery			
HR (bpm)	135.0 ± 12.2	133.7 ± 15.3	0.509
SBP (mmHg)	79.7 ± 6.6	80.8 ± 8.0	0.294
CI (L/min/m^2^)	2.68 ± 0.52	2.63 ± 0.77	0.607
SVRI (dyne s/m^2^ cm^5^)	1676.0 (1435.0, 2113.0)	1624.0 (1338.5, 1974.0)	0.578
dp/dt (mmHg/ms)	0.74 ± 0.24	0.68 ± 0.27	0.234
CCE (units)	− 0.11 (− 0.36,0.10)	− 0.34 (− 0.57,0.02)	0.002
72 h after surgery			
HR (bpm)	129.6 ± 14.2	128.8 ± 13.6	0.707
SBP (mmHg)	82.0 ± 7.9	80.1 ± 8.8	0.120
CI (L/min/m^2^)	2.99 ± 0.47	2.77 ± 0.56	0.01
SVRI (dyne s/m^2^ cm^5^)	1625.8 ± 460.4	1612.2 ± 487.5	0.861
dp/dt (mmHg/ms)	0.81 (0.60, 0.99)	0.76 ± 0.25	0.199
CCE (units)	0.08 (− 0.15, 0.21)	− 0.10 (− 0.42, 0.13)	0.001

Data are means ± standard deviation (SD) or medians [Q1, Q3] for continuous variables and number of subjects (*n*) and percentage (%) for categorical variables

*HR* heart rate, *SBP* systolic blood pressure, *CI* cardiac index, *SVRI* systemic vascular resistance index, *dp/dt*_*MAX*_ maximum pressure gradient, *CCE* cardiac cycle efficiency, *bpm* beats per minute

Exploratory subgroup analysis results were shown in Additional file 1: Table S7. However, there were no significant treatment-by-subgroup interactions with respect to RACHS, cross-clamp time, CPB time, or VIS. There was an observed effect on LCOS incidence between the two treatment groups in the subgroup of patients aged 1 to 6 months. Univariate and multivariate analyses confirmed an association between LCOS and levosimendan (*p* = 0.037) as shown in Additional file 1: Table S8 and Table 5.

**Table 5:** Baseline factors predictive of LCOS incidence based upon a multivariate logistic regression analysis

Variable	OR	95% CI	*p* value
Randomization to levosimendan	0.38	0.15–0.94	0.037
Age	0.98	0.94–1.02	0.282
RACHS	–	–	0.001

Per the “10 events per variable” principle, this multivariate logistic regression model incorporated the most relevant variables related to levosimendan randomization, age, and RACHS and for 1-month increase

*OR* odds ratio, *BMI* body mass index, *CI* confidence interval, *CPB* cardiopulmonary bypass, *RACHS* Risk Adjustment for Congenital Heart Surgery

With respect to safety outcomes, refractory hypotension rates were 2.1% (2/94) and 2.2% (2/93) in levosimendan and placebo groups, respectively, while rates of arrhythmias were 4.26% (4/94) and 3.23% (3/93), respectively. No significant differences were detected with respect to hepatic or renal function variables between the groups, shown in Additional file 1: Table S6.

### Pharmacokinetics

**Fig. 3:**
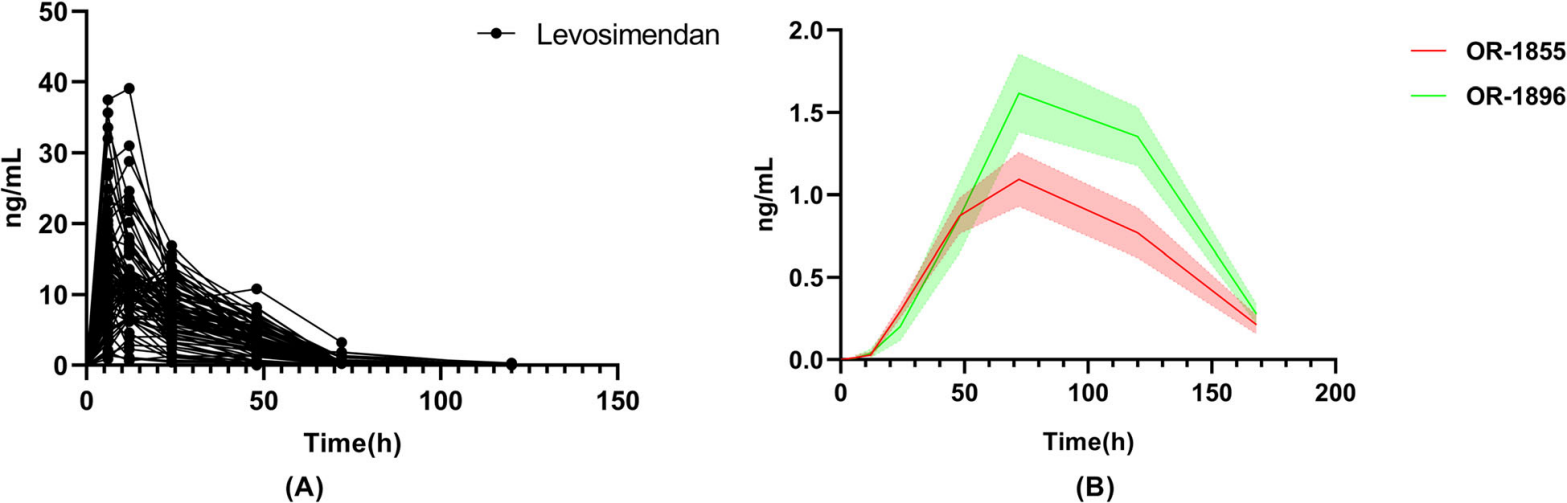
Pharmacokinetics of levosimendan and its metabolites during and after infusion (statistics all patients including infusion duration < 24 h, 24–48 h, and 48 h). **a** Pharmacokinetics of levosimendan. **b** Pharmacokinetics of levosimendan metabolites

Levosimendan and metabolite concentrations at different time points were summarized in Fig. 3. Plasma levosimendan concentrations at 6, 12, 24, 48, 72, and 120 h after initiating the 48-h infusion were 11.61 (7.89, 16.90), 11.0 (8.14, 13.6), 7.17 (4.72, 10.11), 3.43 (2.09, 5.34), and 0.22 (0, 0.32) ng/mL, respectively. The levosimendan terminal half-life was 17.47 h, with the mean peak concentration of 14.94 ± 7.51 ng/mL having been reached 10.21 ± 6.35 h after infusion initiation. The mean AUC_0−*t*_ was 401.13 ± 186.44 h ng/mL. Levosimendan pharmacokinetic parameters were summarized in Additional file 1: Table S9.

## Discussion

This study was the first trial using continuous invasive hemodynamics and pharmacokinetics in order to explore the efficacy and safety of prophylactic levosimendan in pediatric patients undergoing cardiac surgery. We observed no significant benefit from levosimendan administration with respect to LCOS incidence rates, although there was a favorable association with a lower hazard ratio to 0.46 relative to placebo. Levosimendan treatment also failed to have any significant impact on 90-day mortality or on the duration of ICU or hospital stay. However, CCE values in levosimendan treated-patients were higher, consistent with improvements to the ability of the cardiovascular system to maintain homeostasis following treatment. Multivariate logistic regression analyses confirmed the effects of levosimendan on LCOS.

Previous clinical studies with levosimendan have suggested that this drug offers a direct benefit to cardiac function in pediatric trials. Ricci et al. found that levosimendan treatment was associated with a LCOS incidence of 37% as compared with a 61% incidence in placebo-treated patients in a population of RACHS 3–4 neonates [18]. Similarly, Amiet et al. demonstrated that the use of levosimendan as a rescue therapy was associated with significant reductions in plasmatic lactate, VIS reductions, and increases in diuresis and SvO_2_ [17]. However, three recent randomized, placebo-controlled, multicenter studies: LICORN [19], CHEETAH [20], and LEVO-CTS [21], failed to demonstrate any impact of levosimendan administration on a broader mortality endpoint. A meta-analysis also confirmed this neutral effect in pediatric patients undergoing surgical treatment of CHD [22]. Our present results were not entirely consistent with these previous prospective clinical trials. We did observe a non-significant trend towards decreased LCOS incidence in levosimendan-treated patients relative to placebo controls (10.6% versus 19.4%), and no benefits in the outcomes such as mortality or duration of hospital/ICU stay following levosimendan administration. However, the subgroup analysis of patients aged 1 to 6 months indicated the efficacy of levosimendan, the use of which was associated with a significant reduction in LCOS. Furthermore, baseline factors predictive of LCOS revealed that levosimendan administration was an independent predictor of LCOS, and this was just as important as was RACHS classification. Although we were unable to rule out the null hypothesis, we felt that this nonetheless still suggested that levosimendan might be a promising and effective inodilator drug for use in cardiac surgery.

Prediction of cardiac function parameters is primarily based upon invasive hemodynamic findings, biomarker findings, and echocardiography findings [23]. Such invasive measurements are not routinely performed in children, and as such, LCOS is usually defined based upon objective symptoms including tachycardia, metabolic acidosis, low SBP, oliguria, and poor perfusion with increasing core-peripheral temperature gap [24]. However, basic clinical symptoms may lead to an inaccurate diagnosis of LCOS, potentially explaining why the incidence of LCOS in our study was significantly lower than in previous studies. Relative to more commonly used methods for CO/CI estimation, PRAM simplifies the implementation, making it of significant value for monitoring LCOS and associated deterioration in pediatric patients [25–27]. In our study, we found that HR and SAP were the least sensitive to LCOS, while CI and biomarkers also fell significantly and required an extended period of time to recover. These values, however, may be modulated by other organs to yield false-positive results, in addition to having the potential to be insufficiently sensitive to changes in acute hemodynamic parameters. Interestingly, CCE, after levosimendan treatment, deviated significantly from that in patients in the placebo group. Scolletta et al. found CCE was of prognostic relevance with cardiac surgery, correlated well with NT-proBNP levels [28]. With respect to the value of CCE in more detail (as reported in Additional file 1), our results suggested that CCE offered a means whereby it was possible to identify suitable cardiac function after cardiac surgery, in addition to revealing that levosimendan exerted a positive inotropic effect, normalizing vasodilation and energy expenditure and thereby aiding the recovery of pediatric patients.

At present, levosimendan is not routinely administered to pediatric patients. The LeoPARDS trial found that a 0.2-μg/kg/min infusion dose linked to increased hypotension and arrhythmia incidence [29]. In the CHEETAH trial, without a loading dose and with an average of 0.07-μg/kg/min dose, very low hypotension and arrhythmias incidence were observed [20]. Amiet et al. found levosimendan to be well tolerated in pediatric patients after cardiac surgery [17]. Higher doses of levosimendan have the potential to achieve greater hemodynamic effects at the cost of more pronounced vasodilatation and consequent hypotension, and as such, these results suggest that it is rationale to omit a loading dose, with individual determinations being made with regard to the maintenance infusion dose (0.05–0.2 μg/kg/min). With respect to the optimal levosimendan dosing duration, a range of durations from 24 to 72 h have been reported in pediatric clinical settings [17, 30]. Physicians in our center determined that levosimendan should be administered as early as possible post-surgery, with a 48-h infusion period serving to strike a balance between the potential for reduced LCOS and potential adverse events. We observed no differences in adverse events between the levosimendan and placebo groups, consistent with most previous reports suggesting that levosimendan was well tolerated in pediatric patients.

Another goal of this study was to explore levosimendan and its metabolite plasma concentrations over time in pediatric patients. Surprisingly, we did not detect any plateau in levosimendan concentration during the infusion period, with a very rapid decline in these concentrations in contrast to the results from previous studies [31]. There may be several explanations for this unique pharmacokinetics. For one, physicians had the ability to adjust the levosimendan regimen delivered to patients according to adverse events. Variations in infusion rates might have led to reduced plasma drug concentrations and rapid drug elimination. Second, how levosimendan interacts with other drugs is not well documented. Routine complement blood volume and diuresis treatments are likely to disrupt drug concentrations [32]. The excessive urine production of patients may have led to more rapid levosimendan excretion. Third, the pathological state of patients following cardiac surgery can impact drug pharmacokinetics. Levosimendan has been shown to be excreted into the small intestine, where it is reduced mainly by intestinal bacteria to OR-1855 [33]. Antibiotic administration may result in intestinal dysbiosis that is more common in pediatric patients. Alternatively, mechanical ventilation and long-term immobilization may have led to gastrointestinal dysfunction. In addition, as 97–98% of levosimendan binds to plasma proteins, mainly albumin, significant postoperative reductions in albumin levels may result in decreased drug binding and more rapid elimination from circulation. Fourth, age-related variables such as the percentage of body water and the immaturity of metabolic pathways affect drug pharmacokinetics [31]. Lastly, no pharmacokinetic data in Asians and other ethnic groups except Caucasians and Blacks are so far available. It was proved that there was a relationship between genetic phenotypes and the metabolism of levosimendan [34]. These factors suggest that a 0.025–0.1-μg/kg/min infusion is safe for pediatric patients but results in lower-than-expected plasma concentrations that may fail to achieve maximal clinical efficiency. In addition, while the majority of the clinical activity of levosimendan is attributable to its active metabolite OR-1896 with a long half-life (about 81 h) [35], rapid reductions in drug levels shorten the long-term clinical efficacy. As such, pediatric patients may achieve better clinical outcomes from either a longer infusion period or repeated infusions [36].

There are certain limitations to the present trial. For one, the thermodilution method is regarded as the gold-standard technique in hemodynamically stable subjects. Although there are many studies demonstrating the efficient use of PRAM when the patient vascular tone is affected [37], the accuracy of PRAM requires further exploration. Furthermore, the lower rate of LCOS than the power calculations predicted may have limited the statistical power of our study. As a result, the enrollment of more patients may be needed in order to overcome this low LCOS incidence. Finally, considering the low body weight of pediatric patients and the need to strictly control supplemental fluid administration following cardiac surgery, we prepared levosimendan in a 45-mL volume of glucose solution rather than the recommended 250 mL. At such high concentrations, the stability of the molecule is not guaranteed and precipitation may occur. This formulation thus necessitates further assessment and confirmation.

## Conclusions

In summary, we conclude that levosimendan is a safe and promising effective inodilator for prophylactic administration in pediatric patients undergoing cardiac surgery. However, the magnitude of the effect of this agent is not as large as previously thought, and our trial could not rule out the null hypothesis. Further in-depth assessment of the utility of levosimendan will require additional trials in order to better study the pharmacokinetics of appropriate doses to balance its hemodynamic effects and adverse events. Future studies need also seek to minimize the impact of CHD category and other patient-associated variables on study outcomes through appropriate methodological variations.

## Supplementary information

**Additional file 1: **Supplemental description of methods and results, including RACHS method, Placebo preparation, Clinical Management, Details of CCE, **Tables S1-S9** and **Figure S1**.

## Data Availability

The datasets used and/or analyzed during the current study are available from the corresponding author on reasonable request.

## Abbreviations

LCOS: Low cardiac output syndrome
OR: Odds ratio
CI: Confidence interval
CHD: Congenital heart disease
CPB: Cardiopulmonary bypass
ICU: Intensive care unit
RACHS: Risk Adjustment for Congenital Heart Surgery
PRAM: Pressure recording analytical method
CI: Cardiac index
SVRI: Systemic vascular resistance index
dp/dt_MAX_: Maximum pressure gradient
CCE: Cardiac cycle efficiency
VIS: Vasoactive-Inotropic Score
HPLC: High-performance liquid chromatography
AKI: Acute kidney injury
BSA: Body surface area
BMI: Body mass index
HR: Heart rate
MAP: Mean arterial pressure
