# Supplementary material for: Prophylactic use of levosimendan in pediatric patients undergoing cardiac surgery: a prospective randomized controlled trial
Source: Crit Care. 2019 Dec 30;23:428. doi: 10.1186/s13054-019-2704-2 (PMC6937718; doi:10.1186/s13054-019-2704-2)
Supplement: Supplementary file 1 — Additional file 1: Supplemental description of methods and results, including RACHS method, Placebo preparation, Clinical Management, Details of CCE, Tables S1-S9 and Figure S1. [file 13054_2019_2704_MOESM1_ESM.docx]

**Additional file 1**

This appendix has been provided by the authors to give readers additional information about their work.

**Prophylactic use of Levosimendan in pediatric patients undergoing cardiac surgery: a prospective randomized controlled trial**

Anbiao Wang^1#^, Chaomei Cui ^1#^, Yiou Fan ^3^, Jie Zi ^1^, Jie Zhang^1^, Guanglai Wang ^1^, Fang Wang^1^, Jun Wang^4^, Qi Tan ^1,2*^

**TABLE OF CONTENTS**

Details RACHS method and enrolled patients page 3

Placebo preparation page 3

Clinical Management page 4

Details the cardiac cycle efficiency (CCE) page 4

Adverse events page 5

**Table S1.** Study eligibility criteria page 7

**Table S2.** Hypotension and tachycardia vital signs page 8

**TableS3.** Myocardial enzymogram analysis page 9

**Table S4.** Arterial blood gas analysis page 10

**Table S5.** VIS analysis page 11

**Table S6.** Hepatic and renal function page 12

**Table S7.** Explorative subgroup analyses of LCOS incidence page 13

**Table S8.** Univariate analysis of association between baseline variables and the incidence of LCOS. page 14

**Table S9.** Pharmacokinetics of Levosimendan in plasma page 15

**Figure S1.** Study drug infusion guide. Page 16

**Supplementary References** page 17

**Details RACHS method and enrolled patients**

In order to properly detect those patients most likely to benefit from a given preventative or interventional approach, proper risk stratification is essential. The Children’s Hospital Boston team therefore developed the Risk Adjustment for Congenital Heart Surgery (RACHS) approach with input from a panel of 11 prominent pediatric cardiologists and cardiac surgeons. This method utilized a combination of clinician judgement, as well as data from 2 national databases, in order to sub-divide 207 surgical procedures into categories of 1 to 6 based upon the risk of mortality [1,2]. The discriminatory power of this model has been found to be further increased via incorporating three additional factors: age, prematurity, and noncardiac congenital structural abnormalities. The RACHS-1 method can be applied in nearly all cases of surgery for congenital heart disease (CHD) and can provide risk adjustment for comparisons of in-hospital mortality in childhood. In the categories of 1 to 6, the greater the score, the higher the risk associated with the procedure. Full inclusion/exclusion criteria are shown in Table S1.

**Placebo preparation**

Control patients were administered a solution containing a mixture of vitamins designed to be identical in color to the Levosimendan solution. This same approach has been used in over 60 different randomized trials of Levosimendan [3].

This Placebo has never been associated with any relevant cardiovascular effects, and the total vitamin dose administered was below standard vitamin supplementation dose recommendations, making them unlikely to influence and pertinent hemodynamic or cardiovascular parameters [4].

**Clinical Management**

With the exception of the defined infusion protocol, all other aspects of patient care were conducted based upon the discretion and judgement of an attending physician. The use of any inotropic or vasoconstrictive agents (epinephrine, norepinephrine, dobutamine, dopamine, etc.) to support the circulatory system was at the discretion of this physician [5]. All pediatric patients received a standardized perioperative antibiotic prophylactic regimen [6]. The same ERAS procedures were used for patients in both groups [7]. The update 2018 American Heart Association on pediatric advanced life support guidelines were followed in all respects when conducting cardiopulmonary resuscitation and emergency cardiovascular care in pediatric patients [8]. In addition, all patients received nutritional therapy in line with the European Society for Clinical Nutrition and Metabolism Standard Operating Procedures [9]. Open-label use of Levosimendan was not permitted as there are currently no guidelines recommending its perioperative use.

**Cardiac cycle efficiency (CCE)**

The novel cardiac cycle efficiency (CCE) indicator describes hemodynamic performance in terms of energy expenditure: systolic energetic performance/total energetic expenditure of the heartbeat. Therefore, CCE may theoretically offer prognostic value. CCE is calculated as follows [10]:

K(t) is defined as the ratio of pressures P_e_ (mean pressure expected) and P_m_ (mean pressure measured).

A power function W(t)_sys_ and W(t)_beat_ is defined as follows:

W(t)_sys_=$\sum_{n=1}^{n} *$($\sum_{i=2n-1}^{n} \frac{p_{i}}{t_{i}}\pm\sum_{j=2n}^{n} \frac{p_{i}}{t_{i}}$)

W(t)_beat_=$\sum_{n=1}^{n} *$($\sum_{i=2n-1}^{n} \frac{p_{i}}{t_{i}}\mp\sum_{j=2n}^{n} \frac{p_{i}}{t_{i}}$)

where: P_i_, P_j_: pressure in the i-th , j-th points of the waveform during systole; t_i_, t_j_ : time at P_i_ , P_j_.

The power W(t)_sys_, W(t)_beat_ and K(t) can be used to measure the “numerical magnitude” and express the overall status of the cardio-vascular system during a single heartbeat:

CCE=$\frac{W_{sys}}{W_{beat}}$k_(t)_

**Adverse events**

Associations between drug dosing and adverse events including headache, hypotension, hypokalemia, and arrhythmia were assessed. Furthermore, Aspartate Aminotransferase (AST), alanine aminotransferase (ALT), total bilirubin in serum (TBIL), albumin (ALB), blood urea nitrogen (BUN), and creatinine (CREA) were used to monitor hepatic and renal function, shown in Table S5.

Tachycardia is the type of arrhythmia that most often affects pediatric patients. The criteria defining it are age-dependent, with a definition of 3+ consecutive beats at > 25% the normal sinus rate [11]. In this trial, supraventricular tachycardia, atrioventricular nodal reentrant tachycardia, atrioventricular reentrant tachycardia, atrial flutter/atrial fibrillation/intra-atrial reentrant tachycardia, and ventricular arrhythmias were all incorporated into the arrhythmia category.

Hypotension was defined as a systolic blood pressure (SBP) < the 5^th^ percentile for normal individuals of comparable age, sex, and height based upon vasopressor infusion without hypovolemia [12].

In light of the shock recognition guidelines produced by the American College of Critical Care Medicine Clinical Practice Guidelines, our center produced a table of basic vital signs which could be used to detect hypotension and tachycardia following cardiac surgery, shown in **Table S2** [13].

A total of 11 Levosimendan group pediatric patients had a < 48 h infusion time, with 6 due to infusion-related adverse events (3 tachycardia, 1 ventricular tachycardia, and 2 refractory hypotension). The remaining 5 patients underwent interruptions for other reasons (1 insufficient surgical correction, 2 hypovolemia, 1 septicemia).

A total of 7 Placebo group pediatric patients had a < 48 h infusion time, with 5 due to infusion-related adverse events (2 tachycardia, 1 ventricular tachycardia, and 2 refractory hypotension). The remaining 2 patients underwent interruptions due to hypovolemia.

**Table S1**. Study eligibility criteria

Diagnosis and main criteria for inclusion and exclusion:

Pediatric patients meeting the following criteria were enrolled:

Primary inclusion criteria:

• Parents provided written, signed, and dated informed consent

• Age ≤48 months

• RACHS Category 2 - 5 operation

Primary exclusion criteria:

• Known hypersensitivity to Levosimendan or any other excipient

• Systemic infections of any kind within 72 h before surgery

• Undergoing emergency operation

• Weight < 2.0 kg

•Severe arrhythmia or hypotension

• Hematologic, pulmonary, hepatic, renal, immunologic, central nervous system, or endocrine system disorders that made patients unsuitable for the present study

• The presence of a mechanical assist device (extracorporeal membrane oxygenation [ECMO]) placed prior to surgery

• cardiac output of ≤2.2 L/min/m² prior to surgery

• Patients had been previously administered Levosimendan within 15 d before starting study drug administration

• Patients had received any other investigational medicinal products within 30 days, or were enrolled in any other interventional trials with the potential to interact with Levosimendan

• Family members of the employees of the study center or associated investigators or those directly involved in the study

**Table S2.** Hypotension and tachycardia vital signs

| Age | Heart rate(bpm) | Systolic BP (mmHg) |
| --- | --- | --- |
| 0 -1 month | >185 | ˂70 |
| 1-3months | >180 | ˂75 |
| 3months-1years | >170 | ˂75 |
| 1-2years | >160 | ˂70+(age in y×4) |
| 2-4years | >140 | ˂70+(age in y×4) |

Note: bpm: beats per minute; y: year.

**Table S3.** Myocardial enzymogram analysis

| Outcome | Levosimendan Group (n=94) | Placebo Group (n=93) | *P* value |
| --- | --- | --- | --- |
| Before surgery |  |  |  |
| TNT (pg/mL) | 21 (13.0,36.8) | 23.0 (13.5,41.5) | 0.513 |
| CK-MB (ng/mL) | 2.35±0.94 | 2.31±0.71 | 0.759 |
| MYO (ng/mL) | 21.0 (21.0,21.0) | 21.0 (21.0,21.0) | 0.655 |
| NT-proBNP (pg/mL) | 236.0 (105.2,621.3) | 412.0 (130.4,1171.0) | 0.178 |
| 24 hours After surgery |  |  |  |
| TNT (pg/mL) | 1239.0 (751.2,1795.5) | 1098.0 (728.3,2085.0) | 0750 |
| CK-MB (ng/mL) | 29.6 (19.7,48.8) | 28.1 (18.0,48.1) | 0.542 |
| MYO (ng/mL) | 76.7 (51.8,99.8) | 81.4 (57.6,126.1) | 0.227 |
| NT-proBNP (pg/mL) | 7291.2 (4286.8,15833.8) | 5035.3 (2717.1,14073.5) | 0.053 |
| 48 hours After surgery |  |  |  |
| TNT (pg/mL) | 876.7 (560.0,1305.8) | 1059.0 (570.2,1515.5.9) | 0.396 |
| CK-MB (ng/mL) | 8.7 (5.2,11.5) | 8.1 (5.1,18.3) | 0.339 |
| MYO (ng/mL) | 21.0 (21.0,33.1) | 28.7 (21.0,44.5) | 0.092 |
| NT-proBNP (pg/mL) | 3424.0 (2025.5,5584.5) | 3867.0 (1916.5,7382.0) | 0.094 |
| 72 hours After surgery |  |  |  |
| TNT (pg/mL) | 839.6 (547.6,1231.3) | 992.0 (477.5,1573.3) | 0.280 |
| CK-MB (ng/mL) | 3.40 (2.66,5.25) | 3.91 (2.59,6.13) | 0.352 |
| MYO (ng/mL) | 21.0 (21.0,21.0) | 21.0 (21.0,21.0) | 0.025 |
| NT-proBNP (pg/mL) | 3374.5 (1913.3,5554.0) | 3140.0 (1690.8,8219.8) | 0.757 |
| 96 hours After surgery |  |  |  |
| TNT (pg/mL) | 742.6 (358.0,1226.0) | 788.1 (357.0,1350.0) | 0.913 |
| CK-MB (ng/mL) | 2.32 (1.81,3.54) | 2.60 (1.91,3.72) | 0.339 |
| MYO (ng/mL) | 21.0 (21.0,21.0) | 21.0 (21.0,21.0) | 0.092 |
| NT-proBNP (pg/mL) | 2548.0 (1457.0,3643.0) | 2697.0 (1101.0,7232.0) | 0.094 |

Data are means ± standard deviation (SD) or medians [Q1, Q3] for continuous variables, and number of subjects (n) and percentage (%) for categorical variables.

**Table S4.** Arterial blood gas analysis.

| Outcome | Levosimendan Group (n=94) | Placebo Group (n=93) | *P* value |
| --- | --- | --- | --- |
| Before surgery |  |  |  |
| PaO_2_ (mmHg) | 120.0 (65.8,169.3) | 124.0 (68.0,190.5) | 0.212 |
| PaCO_2_ (mmHg) | 37.0±7.8 | 36.7±7.9 | 0.844 |
| Lac (mmol/L) | 1.8 (1.2,2.8) | 1.9 (1.4,2.9) | 0.431 |
| 24 h After surgery |  |  |  |
| PaO_2_ (mmHg) | 78.0 (59.0,111.3) | 82.0 (63.0,119.5) | 0.571 |
| PaCO_2_ (mmHg) | 36.7±8.2 | 37.6±10.5 | 0.514 |
| Lac (mmol/L) | 1.2 (0.8,1.6) | 1.20 (0.8,1.6) | 0.196 |
| 48h After surgery |  |  |  |
| PaO_2_ (mmHg) | 88.0 (64.8,127.3) | 88.0 (68.0,119.5) | 0.991 |
| PaCO_2_ (mmHg) | 39.0±6.2 | 37.7±6.2 | 0.169 |
| Lac (mmol/L) | 0.7 (0.5,0.9) | 0.8 (0.6,1.0) | 0.068 |
| 72h After surgery |  |  |  |
| PaO_2_ (mmHg) | 89.2 (73.0,114.8) | 93.3 (73.5,103.5) | 0.875 |
| PaCO_2_ (mmHg) | 39.0±6.6 | 39.7±6.7 | 0.843 |
| Lac (mmol/L) | 0.7 (0.4,0.9) | 0.8 (0.5,0.8) | 0.056 |

Note: Data are means ± standard deviation (SD) or medians [Q1, Q3] for continuous variables

**Table S5.** VIS analysis.

| Outcome | Levosimendan Group (n=94) | Placebo Group (n=93) | *P* value |
| --- | --- | --- | --- |
| 2 h after surgery | 8 (6,10) | 9 (6,12) | 0.301 |
| 1 d after surgery | 6 (4,8) | 6 (5,10) | 0.237 |
| 2 d after surgery | 5 (3,6) | 5 (3,6) | 0.268 |
| 3 d after surgery | 3 (1.75,5) | 4 (2,6) | 0.097 |

Note: Data are medians [Q1, Q3] for continuous variables

VIS: Vasoactive-Inotropic Score

**Table S6.** Hepatic and renal function

| Outcome | Levosimendan Group (n=94) | Placebo Group (n=93) | *P* value |
| --- | --- | --- | --- |
| Before surgery |  |  |  |
| AST (U/L) | 38.5 (32.0,54.3) | 40.0 (33.0,49.0) | 0.808 |
| ALT (U/L) | 16.0 (12.0,31.3) | 16.0 (12.0,26.5) | 0.699 |
| TBIL (μmol/L) | 11.1 (6.9,22.7) | 10.3 (6.7,25.7) | 0.809 |
| ALB (g/L) | 40.3±3.7 | 40.2±5.1 | 0.856 |
| BUN (mmol/L) | 3.5±1.4 | 3.5（2.5,4.4) | 0.253 |
| CREA (μmol/L) | 23.9 (21.5,28.9) | 26.4 (23.2,31.4) | 0.083 |
| 1 day after surgery |  |  |  |
| AST (U/L) | 99.1 (748.3,125.5) | 98.3 (74.3,139.5) | 0.739 |
| ALT (U/L) | 15.1 (12.0,20.0) | 15.5 (13.0,21.0) | 0.493 |
| TBIL (μmol/L) | 22.3 (13.1,39.8) | 24.1 (13.8,51.8) | 0.571 |
| ALB (g/L) | 40.8±6.7 | 40.6±5.0 | 0.811 |
| BUN (mmol/L) | 7.1 (5.6,8.7) | 6.9 (5.6,9.4) | 0.366 |
| CREA (μmol/L) | 29.0 (25.1,33.7) | 30.6 (25.4,39.2) | 0.109 |
| 3 day after surgery |  |  |  |
| AST (U/L) | 35.3 (29.2,48.4) | 34.3 (25.8,47.6) | 0.312 |
| ALT (U/L) | 15.3 (11.1,21.6) | 14.2 (11.8,21.3) | 0.936 |
| TBIL (μmol/L) | 11.7 (8.7,18.4) | 14.4 (9.6,29.1) | 0.142 |
| ALB (g/L) | 38.9±5.8 | 38.1 (35.1,41.8) | 0.324 |
| BUN (mmol/L) | 6.8 (4.8,8.7) | 6.2 (4.5,8.1) | 0.355 |
| CREA (μmol/L) | 22.9 (19.4,27.1) | 24.7 (21.1,32.1) | 0.086 |

Note: Data are means ± standard deviation (SD) or medians [Q1, Q3] for continuous variables

**Table S7.** Explorative subgroup analyses of LCOS incidence

| Subject-analysis | Levosimendan (n=94) | Placebo (n=93) | OR (95%CI) | *P* value |
| --- | --- | --- | --- | --- |
| 1 RACHS classification* no./total no. |  |  |  |  |
| RACHS 2 | 5/58 | 12/67 | 0.72 (0.52,1.03) | 0.131 |
| RACHS 3 | 3/25 | 3/11 | 0.53 (0.21,1.44) | 0.257 |
| RACHS 4 | 2/10 | 3/13 | 0.93 (0.41,2.12) | 0.859 |
| 2 AGE  months -no./total no. |  |  |  |  |
| ＜1 | 4/6 | 6/13 | 1.30 (0.67,2.42) | 0.413 |
| 1-6 | 2/52 | 8/32 | 0.42 (0.26,0.64) | 0.004 |
| 7-12 | 2/19 | 1/19 | 1.54 (0.32,7.91) | 0.552 |
| ＞12 | 2/17 | 3/29 | 1.06 (0.53,2.24) | 0.882 |
| 3 Cross clamp time minutes- no./total no. |  |  |  |  |
| ＜60 | 9/74 | 12/63 | 0.77 (0.47,1.18) | 0.262 |
| ＞60 | 1/20 | 6/30 | 0.65 (0.46,0.98) | 0.133 |
| 4 CPB time  minutes- no./total no. |  |  |  |  |
| ＜60 | 5/34 | 5/25 | 0.82 (0.41,1.65） | 0.586 |
| 60-90 | 3/33 | 5/33 | 0.77 (0.42,1.41) | 0.452 |
| ＞90 | 2/27 | 8/38 | 0.65 (0.43,0.97） | 0.112 |
| 5 VIS at 2 hours after surgery- no./total no. |  |  |  |  |
| ＜10 | 8/73 | 12/64 | 0.58 (0.26,1.34) | 0.203 |
| ＞10 | 2/21 | 6/29 | 0.46 (0.11,2.13) | 0.290 |

Note: number of subjects (n) for categorical variables.

CPB: cardiopulmonary bypass; RACHS: Risk Adjustment for Congenital Heart Surgery

VIS: Vasoactive-Inotropic Score

*There were only 1 patient in Levosimendan group and 2 patients in Placebo group with a RACHS Classification of 5, therefore, we did not compare differences in RACHS 5 frequencies between two groups.

**Table S8**. Univariate analysis of association between baseline variables and the incidence of LCOS. Any value with p < 0.01 was incorporated into a multivariate logistic regression model.

| Variable | LCOS: YES (n=28) | LCOS: NO (n=159)& | *P* value |
| --- | --- | --- | --- |
| Age (months) | 1.8(0.5,7.5) | 6.0(3.0,13.0) | 0.002 |
| Female sex-no. (%) | 7(25.0%) | 76(47.8%) | 0.026 |
| BMI, kg/m^2^ | 13.9(12.6,15.3) | 15.1(13.4,16.3) | 0.049 |
| CPB time (minutes) | 106(72,125) | 73(55,96) | 0.001 |
| Aortic cross-clamp time (minutes) | 63(31,74) | 41(29,59) | 0.01 |
| RACHS classification  -no. (%)‡ |  |  | ˂0.001 |
| 2 | 10(35.7%) | 115(72.3%) |  |
| 3 | 8(28.6%) | 28(17.6%) |  |
| 4 | 10(35.7%) | 13(8.2%) |  |
| 5 | 0 | 3(2%) |  |
| Hemodynamic variables before surgery |  |  |  |
| CI (L/min/m^2^) | 3.37±0.48 | 3.47±0.45 | 0.410 |
| SVRI (dyne*sec/(m^2^*cm^5^)) | 1113.3±188.2 | 1107.2±163.9 | 0.875 |
| dp/dt (mmHg/msec) | 0.85±0.22 | 0.88±0.21 | 0.558 |
| CCE (units) | 0.35±0.11 | 0.36±0.14 | 0.798 |
| Myocardial enzyme before surgery |  |  |  |
| TNT (pg/mL) | 15（11.78,42.75） | 23（13.89,38.25） | 0.517 |
| Ck-MB (ng/mL) | 2.49（2.1,3.09） | 2.3（1.6,3.0） | 0.207 |
| MYO (Ng/mL) | 21（21,21） | 21（21,21） | 0.739 |
| NT-proBNP (pg/mL) | 1157.75（566,3296） | 287（105,715.7） | 0.095 |
| Atrial blood gas analysis |  |  |  |
| PaO_2_ (mmHg) | 76（62.25,126.5） | 137（71,188.5） | 0.016 |
| PaCO_2_ (mmHg) | 38（32.25,48.75） | 36（30,41） | 0.248 |
| Lac (mmol/L) | 1.8（1.625,2.68） | 1.8（1.2,2.8） | 0.598 |

Data are means ± standard deviation (SD) or medians [Q1, Q3] for continuous variables, and number of subjects (n) and percentage (%) for categorical variables.

BMI: body mass index; CPB: cardiopulmonary bypass; RACHS: Risk Adjustment for Congenital Heart Surgery; CI: Cardiac Index; SVRI: Systemic vascular resistances index cardiac index, dp/dt_MAX:_ Maximum Pressure Gradient; CCE: Cardiac Cycle Efficiency.

&Differences between percent values are given in percentage points, thus potentially not summing to expected values as a consequence of rounding. Other variable differences are in the indicated units.

**Table S9.** Pharmacokinetics of Levosimendan in plasma.

| Parameter | Mean±S.D. | 95% CI |
| --- | --- | --- |
| T_1/2_(hours) | 16.47±9.32 | 14.44-18.49 |
| T_max_(hours) | 10.21±6.35 | 8.79-10.92 |
| C_max_ (ng/mL) | 14.94±7.51 | 13.34-16.57 |
| AUC_0-t_ (hour*ng/mL) | 401.13±186.43 | 362.45-442.81 |
| AUC_0-∞_(hour*ng/mL) | 449.41±195.47 | 406.97-491.82 |

Note: Data are means ± standard deviation (SD) for continuous variables.

T_1/2_: Terminal half-life; T_max_: Time to peak concentration; C_max_: peak concentration in plasma; AUC_0-t_: Area under the curve to the last measurable concentration; AUC_0-∞_: Area under the concentration time curve.

The study drug infusion guide was developed based on LeoPARDS study [14]. The central venous pressure (CVP) was monitored for all patients and was maintained in a 10-15 mmHg range. Reductions in blood pressure were first addressed through reassessment of volume status with appropriate treatment, after which study drug and vasopressor dosages were adjusted as appropriate.


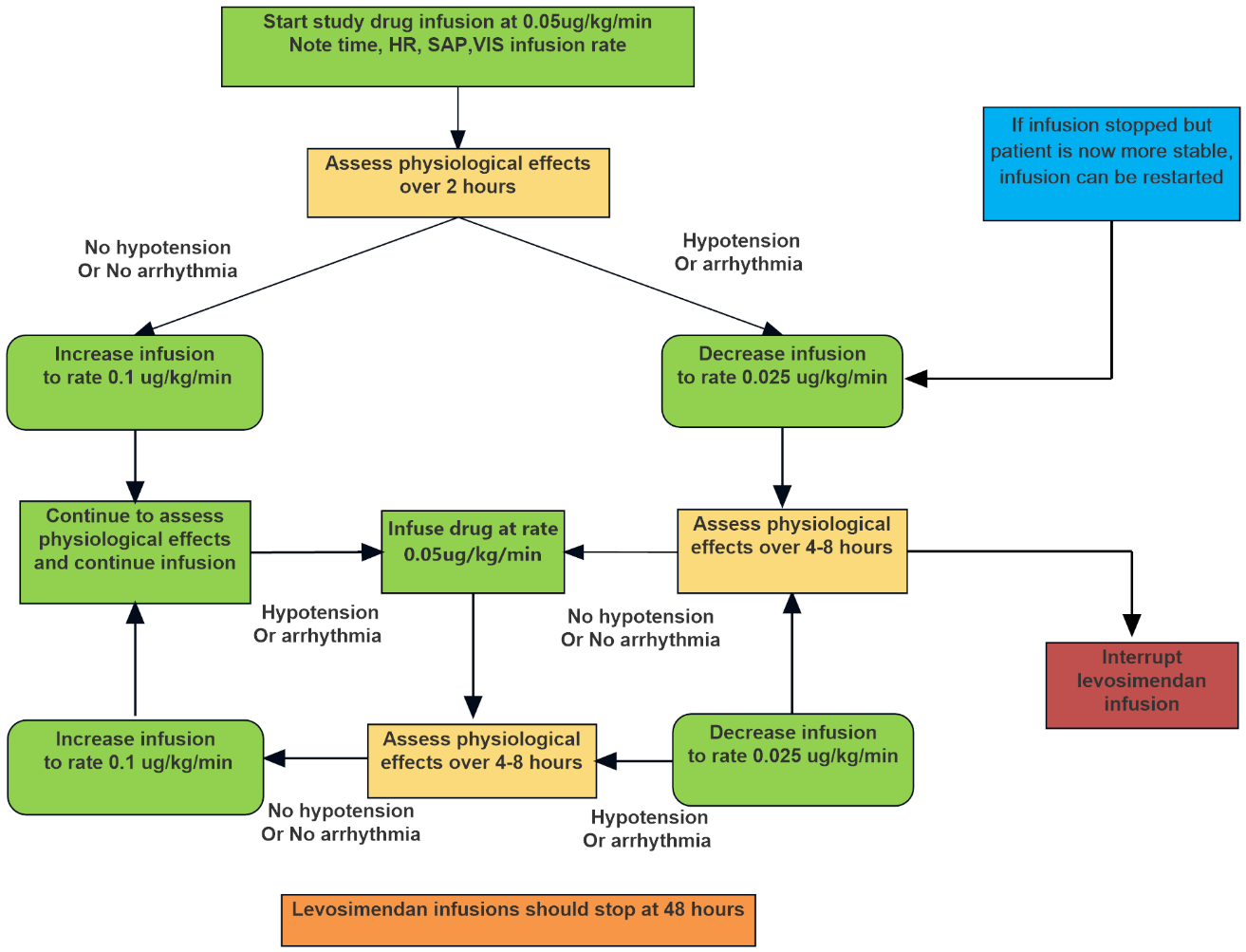


**Fig. S1** Study drug infusion guide. HR: heart rate; SAP: [systolic](javascript:;) [arterial](javascript:;) [pressure](javascript:;); VIS: Vasoactive-Inotropic Score.

**Reference**

1. Brown KL, Rogers L, Barron DJ, Tsang V, Anderson D, Tibby S, et al. Incorporating comorbidity within risk adjustment for UK pediatric cardiac surgery. Ann Thorac Surg. 2017;104:220-6.
2. Jenkins KJ, Gauvreau K, Newburger JW, Spray TL, Moller JH, Iezzoni LI. Consensus-based method for risk adjustment for surgery for congenital heart disease. J Thorac Cardiovasc Surg. 2002;123:110-8.
3. Belletti A, Castro ML, Silvetti S, Greco T, Biondi-Zoccai G, Pasin L, et al. The Effect of inotropes and vasopressors on mortality: a meta-analysis of randomized clinical trials. Br J Anaesth. 2015;115:656-75.
4. DiNicolantonio JJ, Niazi AK, Lavie CJ, O'Keefe JH, Ventura HO. Thiamine supplementation for the treatment of heart failure: a review of the literature. Congest Heart Fail. 2013;19:214-22.
5. Subirana M T, Barón-Esquivias G, Manito N, Oliver JM, Ripoll T, Lambert JL, et al. 2013 update on congenital heart disease, clinical cardiology, heart failure, and heart transplant. Rev Esp Cardiol (Engl Ed). 2014;67:211-7.
6. Murray MT, Corda R, Turcotte R, Bacha E, Saiman L, Krishnamurthy G. Implementing a standardized perioperative antibiotic prophylaxis protocol for neonates undergoing cardiac surgery. Ann Thorac Surg. 2014;98:927-33.
7. Shinnick JK, Short HL, Heiss KF, Santore MT, Blakely ML, Raval MV. Enhancing recovery in pediatric surgery: a review of the literature. J Surg Res. 2016;202:165-76.
8. Duff JP, Topjian A, Berg MD, Chan M, Haskell SE, Joyner BL Jr, et al. 2018 American Heart Association focused update on pediatric advanced life support: An update to the American Heart Association Guidelines for cardiopulmonary resuscitation and emergency cardiovascular care. Circulation. 2018;138:e731-e739.
9. Singer P, Blaser AR, Berger MM, Alhazzani W, Calder PC, Casaer MP, et al. ESPEN guideline on clinical nutrition in the intensive care unit. Clin Nutr. 2019;38:48-79.
10. Romano SM. Cardiac cycle efficiency: a new parameter able to fully evaluate the dynamic interplay of the cardiovascular system. Int J Cardiol. 2012;155:326-7.
11. Drago F, Battipaglia I, Di Mambro C. Neonatal and pediatric arrhythmias: Clinical and electrocardiographic aspects. Card Electrophysiol Clin. 2018;10:397-412.
12. Rosner B, Cook N, Portman R, Daniels S, Falkner B. Determination of blood pressure percentiles in normal-weight children: some methodological issues. Am J Epidemiol. 2008;167:653-66.
13. Davis AL, Carcillo JA, Aneja RK, Deymann AJ, Lin JC, Nguyen TC, et al. American College of Critical Care Medicine clinical practice parameters for hemodynamic support of pediatric and neonatal septic shock. Crit Care Med. 2017;45:1061-93.
14. Gordon AC, Perkins GD, Singer M, McAuley DF, Orme RM, Santhakumaran S, et al. Levosimendan for the prevention of acute organ dysfunction in sepsis. N Engl J Med. 2016;375:1638-48.
